# Nitrogen isotopic composition as a gauge of tumor cell anabolism-to-catabolism ratio

**DOI:** 10.1038/s41598-023-45597-z

**Published:** 2023-11-13

**Authors:** Marietta Straub, Alexandra Auderset, Laurence de Leval, Nathalie Piazzon, Damien Maison, Marie-Catherine Vozenin, Jonathan Ollivier, Benoît Petit, Daniel M. Sigman, Alfredo Martínez-García

**Affiliations:** 1 Institute of Radiation Physics, Lausanne University Hospital and University of Lausanne, Lausanne, Switzerland; 2 Max Planck Institute for Chemistry, 55128 Mainz, Germany; 3 School of Ocean and Earth Science, University of Southampton, Southampton, SO14 3ZH UK; 4 Institute of Pathology, Department of Laboratory Medicine and Pathology, Lausanne University Hospital and University of Lausanne, Lausanne, Switzerland; 5 Radiation Oncology Laboratory/DO/Radio-Oncology/CHUV, Lausanne University Hospital and University of Lausanne, Lausanne, Switzerland; 6 Department of Geosciences, Princeton University, Princeton, NJ 08544 USA

**Keywords:** Biogeochemistry, Cancer

## Abstract

Studies have suggested that cancerous tissue has a lower ^15^N/^14^N ratio than benign tissue. However, human data have been inconclusive, possibly due to constraints on experimental design. Here, we used high-sensitivity nitrogen isotope methods to assess the ^15^N/^14^N ratio of human breast, lung, and kidney cancer tissue at unprecedented spatial resolution. In lung, breast, and urothelial carcinoma, ^15^N/^14^N was negatively correlated with tumor cell density. The magnitude of ^15^N depletion for a given tumor cell density was consistent across different types of lung cancer, ductal in situ and invasive breast carcinoma, and urothelial carcinoma, suggesting similar elevations in the anabolism-to-catabolism ratio. However, tumor ^15^N depletion was higher in a more aggressive metaplastic breast carcinoma. These findings may indicate the ability of certain cancers to more effectively channel N towards growth. Our results support ^15^N/^14^N analysis as a potential tool for screening biopsies and assessing N metabolism in tumor cells.

## Introduction

Natural abundance isotopic studies can provide temporally integrative constraints on the metabolic states and “strategies” of tissues^1–6^. The stable isotopes of nitrogen (N) have been used for decades in biological, ecological, environmental, and geological systems^7–17^. Because of the broad application of such isotopic tracers in these fields, methods for N isotope measurement at natural abundance concentrations have been continuously improved and optimized for sensitivity, and techniques now enable the isotopic characterization of minute quantities of N in a wide diversity of matrices^10, 18–22^. In recent years, interest has risen in the use of natural abundance N stable isotopes in medicine to study cellular metabolism^5, 23–30^.

In cancer cells, which tend to grow rapidly, the scarcity of N can represent an important constraint; conversely, cancer cells can develop the ability to acquire and retain N under these conditions of scarcity^31–33^. For example, in breast cancer cells, Spinelli et al. (2017) showed that tumor cells actively recycle catabolically produced ammonium (NH_4_^+^), normally a toxic by-product to be eliminated from the cellular environment. De- and trans-amination reactions in tissues and organisms generally result in a series of ^15^N-depleted N by-products (e.g., ammonium, urea, and uric acid), which are eliminated from the cellular environment and excreted from the organism^34–36^. The elimination of these ^15^N-depleted N metabolites results in an increase in the ^15^N/^14^N of benign tissues with respect to their N source^7, 37^. Thus, if tumor cells are capable of retaining N for growth, such as through actively recycling catabolic NH_4_^+^, we would expect their ^15^N/^14^N to be lower than the surrounding healthy cells. Consequently, the isotopic difference between benign and cancerous tissue could be used as a diagnostic tool to identify tumor cells on the basis of their altered N metabolism.

In Straub et al. (2021), using murine models of brain and head and neck cancer, we showed that tumor tissue micro-biopsies have a significantly lower ^15^N/^14^N ratio relative to the surrounding benign tissue. Brain tumors (glioblastoma), which are relatively homogeneous and have high tumor cell density (TCD), showed lower ^15^N/^14^N than the surrounding benign tissue. In the case of head and neck cancer, benign tissue was infiltrated by the tumor and vice versa, and the ^15^N/^14^N difference between benign and cancerous tissue was more variable, pointing to the potential importance of tissue heterogeneity in the ^15^N/^14^N in tumor biopsies. A number of other studies have investigated if cancerous tissues are characterized by a lower ^15^N/^14^N than benign tissue in human biopsies^26–29, 38, 39^. However, most of these studies did not measure the ^15^N/^14^N of benign and tumor tissue from the same patient, and the potential influence of tissue heterogeneity and/or tumor cell abundance in the biopsies has not been assessed. These factors have led to ambiguous results across patients and cancer types and have limited the apparent potential of natural abundance ^15^N/^14^N measurements as a tool for identifying tumor cells and for studying their N metabolism.

In the present study, we combine a high-resolution sampling method with a high-sensitivity analytical technique to investigate: (i) if ^15^N/^14^N enables the discrimination of cancerous *versus* benign tissue in human biopsies, (ii) if the magnitude of ^15^N/^14^N depletion in cancer tissues is related quantitatively to tumor cell density as estimated by standard histopathological methods, and (iii) if the degree of ^15^N/^14^N depletion for a given number of tumor cells is consistent across different cancer types.

## Results and discussion

### Subsampling and analysis of cryostat sections

Surgical biopsies sampled as “cancerous tissue” contain variable proportions of stromal components and may be admixed with residual benign tissue, making precise tumor cell sampling difficult. This tissue heterogeneity may explain ambiguous results from the analysis of the N isotopes in large human biopsies^24, 26–28, 38, 39^. Indeed, we demonstrate such a role for heterogeneity in 7 lung and 12 breast cancer biopsies subsampled by fine needle aspiration and/or scalpel (see supplementary information S1). In order to circumvent these complicating factors, we developed a novel approach for the analysis of N isotopes using sequential cryostat sections of primary tumor after surgical removal.

**Figure 1:**
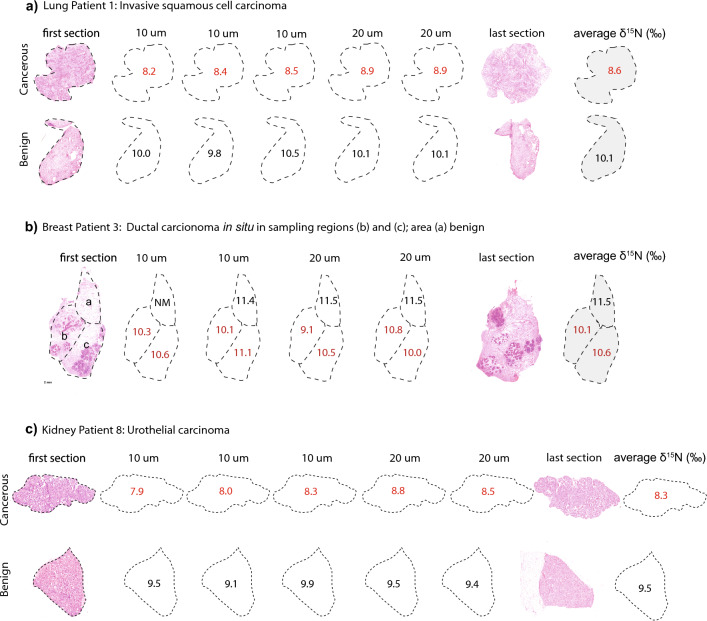
Sampling and N isotopic analysis of serial cryostat sections from cancerous and benign tissues. In this figure, we show the results of nitrogen isotope measurements in representative cryostat sections for each cancer type (lung, breast and kidney); the rest of the sections are shown in SI Figs. S1–S4. Benign and cancer cell-containing samples were obtained (i) from different cryostat sections (e.g., lung and kidney panels (**a**) and (**c**)), or (ii) from different areas in single cryostat sections (e.g., breast panel (**b**)). (**a**) Lung patient 1 presenting an invasive squamous cell carcinoma. (**b**) Breast cancer patient 3 presenting a ductal carcinoma in situ. Sampling area ‘a’ is “pure” benign tissue, ‘b’ and ‘c’ have tumor cell clusters covering about 60% of the sampling area. (**c**) Kidney cancer patient 8, representing a urothelial carcinoma. In all panels of this figure, red numbers correspond to measured δ^15^N (‰ vs. air) values for tissue with cancer cells present, and black numbers correspond to δ^15^N (‰ vs. air) values for “pure” benign tissue. *NM* not measured.

Cryostat sections were prepared by sequentially cutting frozen tissue samples into 4–20 µm-thick slices (Fig. 1 and SI Figs. S1–S4). The first and last sections (of 4 µm thickness) were stained with hematoxylin and eosin (HE) for pathological identification and quantitative estimation of tumor cell density (TCD). This allowed us to identify sections with a relatively homogenous distribution of tumor cells on which to perform N isotopic measurements. Then, the tissue of the intermediate sections (of 10 or 20 µm thickness) was scraped off the cryostat section slide with a scalpel, and the N isotopic composition of each section was determined individually.

We prepared cryostat sections from 4 patients with lung cancer (one squamous cell carcinoma, lung P1; three adenocarcinomas, lung P2–P4), 5 patients with breast cancer (one metaplastic carcinoma, breast P1; 2 ductal carcinomas in situ, breast P3 and P4; and two invasive carcinomas of no special type, breast P2 and P5), and 8 patients with kidney cancer (seven renal cell carcinomas, kidney P1-P7; and one urothelial carcinoma, kidney P8). From each patient, five additional cryostat sections from matched benign tissue were prepared following the same procedure in order to obtain an estimate of the patient’s ^15^N/^14^N baseline (Fig. 1 a and c, and SI Figs. S1–S4). In three cases of breast cancer (patients 3, 4 and 5), the histopathological sections showed that tumor cells were not homogenously distributed (Fig. 1b and SI Fig. S2). In these cases, we adapted our sampling scheme and measured separately regions with high TCD and benign tissue originating from the same cryostat section.

This sampling approach enabled us to directly compare TCD assessed by standard microscopic analysis with the ^15^N/^14^N in the same samples. The unprecedented level of spatial resolution was made possible by a novel high sensitivity method that allows for accurate ^15^N/^14^N analysis of as few as 2000 cells (see method section for details). From here on, ^15^N/^14^N is expressed in terms of δ^15^N, where δ^15^N (in permil, ‰) = ([(^15^N/^14^N)_sample_/(^15^N/^14^N)_air_] − 1)*1000, where atmospheric (“air”) N_2_ serves as the universal isotopic reference.

### Lower δ^15^N in lung and breast tumors

**Figure 2:**
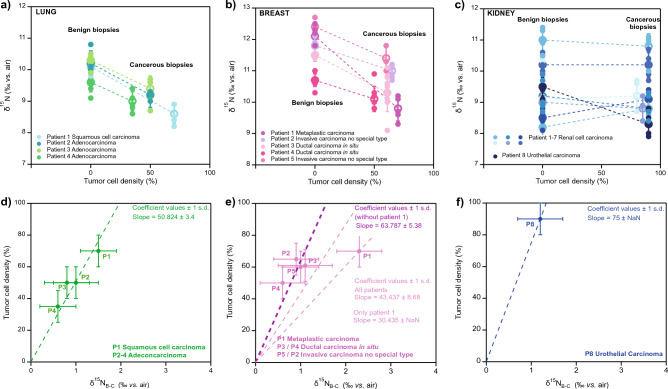
Comparison of nitrogen isotope measurements and histopathological tumor cell density (TCD) estimates for benign and cancer tissues from the same patient. In panels (**a**–**c**), the δ^15^N (‰ vs. air) from the tissue biopsies is plotted against the proportion of tumor cells (i.e., tumor cell density, TCD) for each patient and cancer type. Benign tissue is assigned 0% TCD. (**a**) Lung cancer δ^15^N measurements. We observe statistically significant differences in the δ^15^N of benign and cancerous biopsies for all patients (p < 0.05). (**b**) Breast cancer δ^15^N measurements. Statistically significant differences in the δ^15^N of benign and cancerous biopsies are observed for all patients, with an apparently distinct relationship for the metaplastic carcinoma. (**c**) Kidney cancer δ^15^N measurements. Except for one patient (patient 8), no statistically significant differences in the δ^15^N of benign and cancerous biopsies are recognized in kidney cancer, although the tumor cell density was high (80–90%). Only patient 8 (dark blue), the sole urothelial carcinoma, shows a significant difference in δ^15^N between benign and cancerous tissue. Panels (**d**–**f**) show the linear fits and corresponding coefficient values of the TCD of each tumor type plotted against the δ^15^N difference between benign and cancerous tissue (δ^15^N_B-C_).

In the 4 patients with lung tumors (patients 1–4), cryostat biopsies showed that cancerous tissue exhibited a significantly (p < 0.05) lower δ^15^N compared to the corresponding benign tissue (Fig. 2a, SI Table S1 and SI Fig. S1). The δ^15^N decrease in cancerous tissue with respect to benign tissue was correlated with TCD in all patients (Fig. 2a; note that TCD is by definition 0% for benign tissue). The slopes of the correlations were very similar for the 4 patients analyzed. In contrast, the y-intercept of the regression lines were variable for each patient, indicating different δ^15^N baseline values (i.e. the δ^15^N of benign tissue) for each patient. These baseline differences are to be expected and most likely reflect the different dietary habits of the patients. It is well established that the N isotopic composition of animals reflects their food sources. For example, herbivorous species show lower δ^15^N values than carnivorous species living in the same ecosystem, with δ^15^N increasing about 3–4‰ per trophic level^3, 7, 16, 40–42^. In humans and other omnivorous species, significantly different δ^15^N baselines have been observed (up to ~ 10 ‰), depending on their dietary habits^2, 3, 7, 42–44^. Thus, the 2–4‰ baseline differences observed among the patients falls within the expected range in a modern population.

The remarkable finding of our study is that, independent of their δ^15^N baseline, the level of ^15^N depletion in cancerous biopsies with respect to tumor cell percentage, i.e. the slope of the regression lines in Fig. 2a, was consistent across patients and lung cancer types (i.e., squamous cell carcinoma and adenocarcinoma). If we remove the influence of the patient’s δ^15^N baseline by calculating the difference between benign and cancerous tissue (δ^15^N_B-C_) for each patient, we observe a good correlation between TCD and δ^15^N_B-C_ (Fig. 2d). These findings indicate that, in these types of cancer, δ^15^N_B-C_ is a good predictor of the percentage of tumor cells (i.e. TCD) in a given tissue biopsy, suggesting that δ^15^N_B-C_ could be a valuable tool for cancer diagnosis.

The cryostat sections from breast cancer biopsies show similar results. We observe a statistically significant δ^15^N difference (p < 0.05) between the benign and the tumor biopsies for all patients (Fig. 2b; SI Table S2 and SI Fig. S2). As in the case of lung cancer, the δ^15^N decrease in breast cancerous tissue with respect to δ^15^N of benign tissue was correlated to TCD in all patients. The slopes of the correlations were consistent for patients with invasive carcinoma of no special type (patients 2 and 5) and ductal carcinoma in situ (patients 3 and 4) (Fig. 2e), suggesting similar degrees of N conservation in these tumor types. However, the patient with metaplastic carcinoma (patient 1) exhibited a significantly larger δ^15^N_B-C_ for a similar level of TCD in the biopsy than the other breast cancer types analyzed (Fig. 2e). The larger ^15^N depletion suggests that more of the N supply was retained for growth (rather than being effluxed after catabolism) in metaplastic carcinoma than in the other breast cancer types (see last section). Thus, our data from breast cancer biopsies confirm the potential of δ^15^N_B-C_ for cancer diagnosis and suggest that it may provide additional information on the magnitude of alteration in tumor cell N metabolism.

It should also be mentioned that in the 3 breast cancer patients (patients 3–5) whose biopsy cryostat sections contained both tumor and benign tissue (i.e., on the same section), the level of ^15^N depletion (slope in Fig. 2b) obtained with this sampling approach was indistinguishable from the one obtained with more homogenous tissue sampling (i.e. patient 2). From each of these sections, regions with tumor cells and regions with benign tissue were measured separately (Fig. 1 panel b and SI Fig. S2), and the δ^15^N_B-C_ was calculated by comparing the δ^15^N of adjacent benign and cancerous tissue. These findings highlight the accuracy of our high-resolution sampling and show the potential of the method to identify alterations of cellular N metabolism in samples with variable concentrations of neoplastic cells.

### N isotope evidence for nitrogenous metabolites in renal cell cancer

The kidney cancer results are more complex. The patient presenting urothelial carcinoma (patient 8) showed a significantly (p > 0.05) lower δ^15^N for the cancerous biopsy (Fig. 2c and SI Fig. S4). The level of ^15^N depletion for this patient was consistent with those observed in the lung and breast cancer patients discussed above (Fig. 2f). However, the renal cell carcinoma patients (patients 1 to 7) showed no significant (p > 0.05) δ^15^N differences between cancer and benign biopsies (Fig. 1c; SI Figs. S3 and S4; SI Table S3). We hypothesize that samples from renal cell cancer, which are derived from renal tubular epithelial cells, were “contaminated” with metabolites (likely lower in ^15^N/^14^N) present in the cell structures of both the benign and cancerous tissue, as active renal filtering is taking place in these tissues^30, 45, 46^. This type of N “contamination” does not occur in the case of papillary urothelial carcinoma, which derives from epithelial cells lining the urinary tract from the renal pelvis to the urethra. Unlike renal cells, urothelial cells do not have an active renal filtering function^47, 48^. Therefore, no accumulation of metabolites is to be expected.

### The N isotopes as a gauge for N conservation in cancer

**Figure 3:**
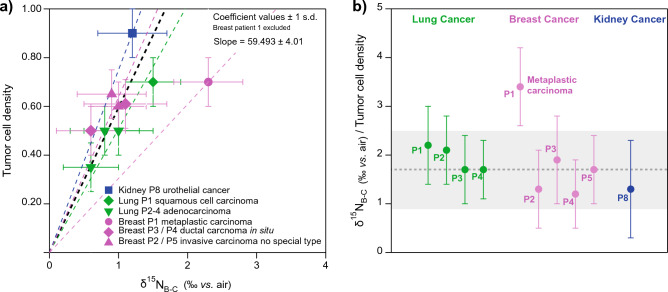
Estimated ^15^N depletion of different cancer cell types. Panel (**a**) shows the linear fits and corresponding coefficient values for the relationship of TCD to the δ^15^N difference between benign and cancerous tissue (δ^15^N_B-C_). Panel (**b**) shows the δ^15^N_B-C_ normalized to TCD, i.e., the level of ^15^N depletion of a cancer cell with respect to a normal cell, for individual patients and cancer types. The grey dashed line indicates the mean of all estimates excluding metaplastic breast carcinoma patient P1, and the grey bar indicates ± 1 s.d.

The level of ^15^N depletion of cancerous tissue with respect to TCD was remarkably consistent among cancer types affecting different organs (Fig. 3a). In Fig. 3b, we calculated the δ^15^N_B-C_ of pure tumor cells by dividing δ^15^N_B-C_ by TCD in each patient and cancer type. The results imply that the level of ^15^N depletion of pure cancer cells is statistically indistinguishable for lung adenocarcinoma and squamous cell carcinoma, breast ductal carcinoma in situ and invasive carcinoma, and kidney urothelial carcinoma. This suggests that the level of N conservation was similar in all these cases, and thus, that these cancer types presented comparable alterations in their cellular N metabolism. In contrast, the δ^15^N_B-C_/TCD ratio observed in the patient with metaplastic breast carcinoma was two times higher than the average of all the other cancer types analyzed (Fig. 3b). This observation implies that the capacity to channel N into growth was significantly greater in this rare form of breast cancer than for all the other cancers.

**Figure 4:**
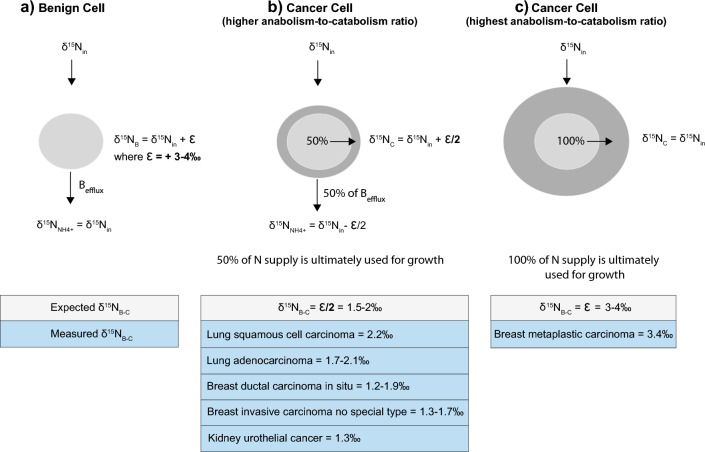
Hypothetical scenarios of N metabolism compared with predicted and observed δ^15^N differences between benign and cancerous tissues from the same organ (δ^15^N_B-C_). (**a**) Benign cell metabolism, with normal ammonium efflux (B_efflux_). (**b**) Scenario of cancerous cells with higher anabolism-to-catabolism ratio, in which 50% of the N supply is incorporated into growth. (**c**) Scenario of cancerous cells with highest anabolism-to-catabolism ratio, in which 100% of the N supply is ultimately used for growth. Table lines with grey background indicate the expected δ^15^N_B-C_ for each scenario assuming an isotope effect (ε) for catabolic ammonium production of 3–4‰. Text with blue background indicates the measured δ^15^N_B-C_ for each cancer type.

Multiple cancers have been shown to minimize the rates of non-anabolic processes^49^. The δ^15^N_B-C_/TCD ratio can be used to estimate the fraction of the N supply directed to tissue growth as opposed to catabolic N loss (i.e., the anabolism-to-catabolism ratio). In this framework, δ^15^N differences between benign and cancerous cells result from the release of low-δ^15^N metabolic ammonium, with higher ammonium loss resulting in a higher cell δ^15^N (Fig. 4). The preference for ^14^N and against ^15^N in a reaction is often quantified as the ε (or “epsilon value” or "isotope effect") of the reaction. ε is defined here as (1-^15^k/^14^k), where ^14^k and ^15^k refer to the ^14^N- and ^15^N-associated rate coefficients for the reaction^50^. Here, the reaction of interest is the catabolic production of ammonium, which typically leads to its efflux from the cell. ε is well approximated as the δ^15^N difference between the substrate (here, the cell N) and the product being produced instantaneously from it (here, the ammonium being produced catabolically in the cell and released into the extracellular environment). In Fig. 4, we compare the N metabolism of benign cells (a) with two hypothetical scenarios for cancer cells, higher anabolism-to-catabolism ratio (b) and highest anabolism-to-catabolism ratio (c), and estimate the expected isotopic changes.

In the case of benign tissue, the δ^15^N of the cell (δ^15^N_B_) is driven higher by the isotope effect of catabolic deamination (ε) and leads to cell δ^15^N elevation compared to its N source (δ^15^N = δ^15^N_in_ + ε) (Fig. 4a). Conversely, the δ^15^N of the N efflux from benign cells (B_efflux_) is roughly equal to the N supply to the cell, given that nearly all of the N supplied to the cell is ultimately catabolized (δ^15^N_NH4_ = δ^15^N_in_). In the case of cancer, it is expected and observed that a malignant cell channels a greater proportion of its N supply to growth rather than eventual excretion, such as by reassimilation of catabolic ammonium^32^. In Fig. 4b, we consider a scenario where 50% of the B_efflux_ is biosynthetically recycled and directed toward growth. In this case, the δ^15^N of the cancerous cell would be less elevated relative to its N inputs than in the case of benign cells (δ^15^N_C_ = δ^15^N_in_ + ε/2). This would result in a δ^15^N difference between benign cells and cancer cells (δ^15^N_B-C_) of ε/2. In Fig. 4c, we consider the end-member case of a fast-growing cancer cell where there is no net catabolic ammonium loss and all N input is eventually included in biosynthesis. In this case, the δ^15^N of the cancer cells would be the same as the N supplied to it (δ^15^N_C_ = δ^15^N_in_). This would result in a δ^15^N difference between benign cells and cancer cells (δ^15^N_B-C_) of ε. It is also possible that cancer cells can assimilate catabolic ammonium produced by other tissues such as neighbouring benign tissue, in which case δ^15^N_B-C_ would be even greater. However, this would predict a lower δ^15^N in the tumor margin than at the tumor core, which is inconsistent with the homogeneity of δ^15^N_B-C_ observed here and in mice tumor models^22^.

Large-scale ecological studies as well laboratory feeding experiments have shown that vertebrate (benign) tissues are typically elevated in δ^15^N by 3–4‰ with respect to their N source (diet)^2, 3, 7, 16, 40–42^. If we assume that this level of isotopic enrichment in the tissue is driven by the excretion of ^15^N depleted catabolic ammonium at a cellular level, the observed ^15^N enrichment in the tissue with respect to its N source can provide an estimate of ε, i.e. 3–4‰*.* Using this value for ε, we calculate the expected cell δ^15^N_B-C_ for the different scenarios presented in Fig. 4: cell δ^15^N_B-C_ values of 1.5–2‰ for scenario b, and 3–4‰ for scenario c. Interestingly, the cellular δ^15^N_B-C_ values measured in this study (i.e. δ^15^N_B-C_/TCD) for lung adenocarcinoma (1.7–2.1‰) and squamous cell carcinoma (2.2‰), breast ductal carcinoma in situ (1.2–1.9‰) and invasive carcinoma (1.3–1.7‰), and kidney urothelial carcinoma (1.3‰) are similar to the expectations of scenario b. This suggests that these cancer cells are ultimately able to channel half of their N supply to growth. In contrast, the δ^15^N_B-C_/TCD value obtained for metaplastic carcinoma (3.4‰) falls within the range of scenario c, suggesting that these cancer cells are characterized by nearly complete retention of their N supply for growth.

Metaplastic carcinoma is an exceedingly rare breast cancer variant that is faster growing and more likely to metastasize than other breast cancer types, and it is more likely to recur after a successful treatment^51, 52^. It is a triple-negative breast cancer with a series of molecular alterations that have been studied as therapeutic targets; however, there are currently no standardized treatment guidelines^52^. Our isotopic data suggest that these types of tumor cells have unique capacity to minimize catabolic N loss. This ability may be crucial to the high growth rates that they can sustain.

### Conclusion

We adopted a novel approach for the analysis of N isotopes using cryostat sections of primary tumor after surgical removal. We analyzed different types of lung, breast and kidney cancer. In lung, breast, and urothelial carcinoma, we found tumor cell density to be inversely correlated with the tissue δ^15^N, with a more pronounced difference in δ^15^N between cancerous and benign tissue being associated with greater proportions of tumor cells as inferred from histopathological examination. The level of ^15^N depletion for a given tumor cell density was remarkably consistent across different types of lung cancer (adenocarcinoma, squamous cell carcinoma), breast cancer (ductal carcinoma in situ and invasive carcinoma) and urothelial carcinoma, suggesting similar alterations in cell N metabolism that allow cancer cells to retain N (e.g., minimize efflux of ^15^N-depleted catabolic metabolites such as ammonium^32^). We estimate that these cancer cell types are able to direct ~50% of their N supply to growth. The ^15^N depletion was two times higher in a patient with a more aggressive form of breast cancer (metaplastic carcinoma), suggesting nearly complete retention of malignant cells’ N supply for their growth. These findings may indicate a link between the ability of certain cancers to prevent catabolic N loss and their capacity to sustain high growth rates. However, this hypothesis requires further testing.

Despite the limited number of patients and cancer types, our data show the potential of natural abundance measurements of N isotopes as a tool to identify alterations in cellular N metabolism that could complement standard histopathological analysis. The δ^15^N difference between benign and cancerous tissue within an organ appears to provide a novel window into the anabolism-to-catabolism ratio of cancers, with a wide range of possibilities to study how this property may relate (and contribute) to the rapid growth and spread of some tumors. The methodology developed in this study of combining microscopic analysis and high-resolution sampling for N isotope analysis using cryostat sections offers a path to test the extent to which these first data apply to other types of cancer and other tumor models. Looking forward, more precise tissue sampling approaches, for example, cell sorting by flow cytometry, may allow a better characterization of the isotopic differences between different types of cancerous and benign cells within the same biopsy. The high sensitivity N isotope methods used here are ideally suited for diverse strategies of dividing biopsy tissue and cells into subsamples in order to further these investigations.

## Methods

### Human tissue biopsies

Tissue biopsies from patients were obtained from the tissue bank of the Institute of Pathology of the University Hospital of Lausanne (CHUV), Switzerland (CER-VD Project ID 2020-02064). Fresh-frozen samples harvested in tumor and adjacent “normal” tissue from surgical specimens had been stored in Tissue-Tek® O.C.T.™ compound (OCT) at − 80 °C. We prepared cryostat section samples from 4 lung cancer patients, 5 breast cancer patients, and 8 kidney cancer patients. Micro-biopsies and/or FNA biopsies, of which results are shown in the SI, were sampled in 7 lung and 12 breast cancer patients.

Cryostat sections prepared for histopathological characterization were stained with hematoxylin and eosin (HE) using a Ventana HE600 automated staining system (Roche Diagnostics), digitalized using a NanoZoomer S60 Digital slide scanner (Hamamatsu Photonics, Japan) at 40 × magnification and evaluated using a digital image viewer system (TM-Microscopy, Telemis, Belgium).

SI Tables S1–S5 report the measurement of δ^15^N associated with the histopathological characterization of tissues from all of lung, breast and kidney patients. Where applicable, approximate tumor cell density (TCD; as % of cell content on the analyzed tissue) was estimated microscopically by the pathologist; these data are included in the supplementary Tables S1–S5.

### Nitrogen isotopic analysis

Nitrogen isotopic analysis was carried out as described in Straub et al. (2021). To summarize briefly: organic N of the tissue samples was converted to nitrate in a basic potassium peroxydisulfate solution^8^, followed by conversion to nitrous oxide (N_2_O) employing denitrifying bacteria that lack an active N_2_O reductase^18^. The obtained N_2_O gaseous product was measured by isotope ratio mass spectrometry^18, 53, 54^. ^15^N/^14^N is expressed in terms of δ^15^N, where δ^15^N (in permil, ‰) = ([(^15^N/^14^N)_sample_/(^15^N/^14^N)_air_] − 1)*1000, where atmospheric (“air”) N_2_ serves as the universal isotopic reference.

### Statistical data analysis

Data from lung cancer patients, breast cancer patients and kidney cancer patients are presented as individual measurements or as measurement means ± 1 standard deviation (s.d.) from different analytical replicates and/or different sampling strategies. Average δ^15^N values of cancerous tissue were compared to the mean value of benign tissue replicates within the same patient with a paired two-tailed Student's test to test for significant difference between cancerous and benign tissue. A p-value of ≤ 0.05 was considered statistically significant. Error propagation was performed where applicable.

### Ethics approval and consent to participate

All procedures were carried out under the license 2020-02064, which was approved by Ethics Authority of the Swiss Canton of Vaud (CER-VD).

### Supplementary Information

Supplementary Information.

## Data Availability

The dataset generated and analyzed for the current study is available in the Supplementary Information (SI Tables S1–S3; SI Figs. S1–S4). Additionally, the file contains the results of complementary analyzed fine needle aspiration biopsies and scalpel-based micro-biopsies, not included in the main manuscript of this study (SI Tables S4, S5; SI Figs. S5, S6).
