# Supplementary material for: Nitrogen isotopic composition as a gauge of tumor cell anabolism-to-catabolism ratio
Source: Sci Rep. 2023 Nov 13;13:19796. doi: 10.1038/s41598-023-45597-z (PMC10643536; doi:10.1038/s41598-023-45597-z)
Supplement: Supplementary file 1 — Supplementary Information. [file 41598_2023_45597_MOESM1_ESM.pdf]

# Supplementary Information: Nitrogen isotopic composition as a gauge of tumor cell anabolism-to-catabolism ratio

M. Straub[1;2]*, A. Auderset[2;3], L. de Leval[4], N. Piazzon[4], D. Maison[4], M.-C. Vozenin[5], J. Ollivier[5], B. Petit[5], D.M. Sigman[6], A. Martínez-García[2*]

[1]Institute of Radiation Physics, Lausanne University Hospital and University of Lausanne, Lausanne, Switzerland
[2]Max Planck Institute for Chemistry, 55128 Mainz, Germany
[3]University of Southampton, School of Ocean and Earth Science, SO14 3ZH Southampton, UK
[4]Institute of Pathology, Department of Laboratory Medicine and Pathology, Lausanne University Hospital and University of Lausanne, Lausanne, Switzerland
[5]Radiation Oncology Laboratory/DO/Radio-Oncology/CHUV, Lausanne University Hospital and University of Lausanne, Switzerland
[6]Department of Geosciences, Princeton University, Princeton, NJ 08544, United States

*Corresponding authors: Straub Marietta and Alfredo Martínez-García

**Emails:** marietta.straub@chuv.ch and a.martinez-garcia@mpic.de

**Content of Supplementary Information**

1. SI Figures S1-S4: Individual cryostat sections of lung, breast and kidney patients
2. Description of measurements done on micro- and FNA biopsies; SI Figures S5-S6
3. Description of SI Tables followed by SI Tables S1-S5

## 1. Individual cryostat sections of lung, breast and kidney patients

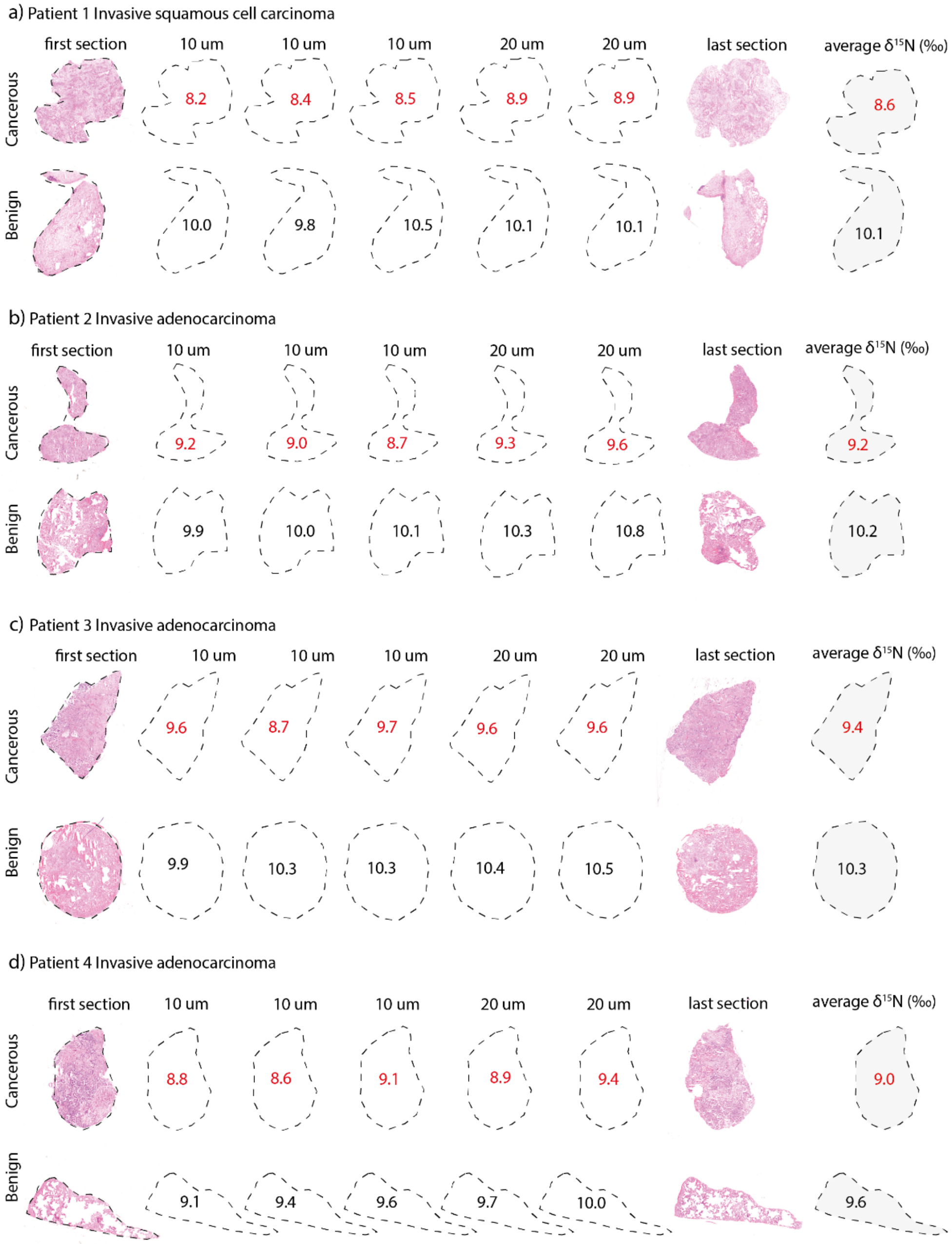


***SI Fig. S1:*** *Lung cryostat sections of patients 1 to 4, top of each panel is cancerous tissue, bottom of each panel is benign tissue. The $\delta^{15}N$ (‰ vs. air) of each cryostat sections is indicated in red writing for cancerous sections and in black for benign sections. On the right of each panel, the average $\delta^{15}N$ (‰ vs. air) of all sections is indicated.*

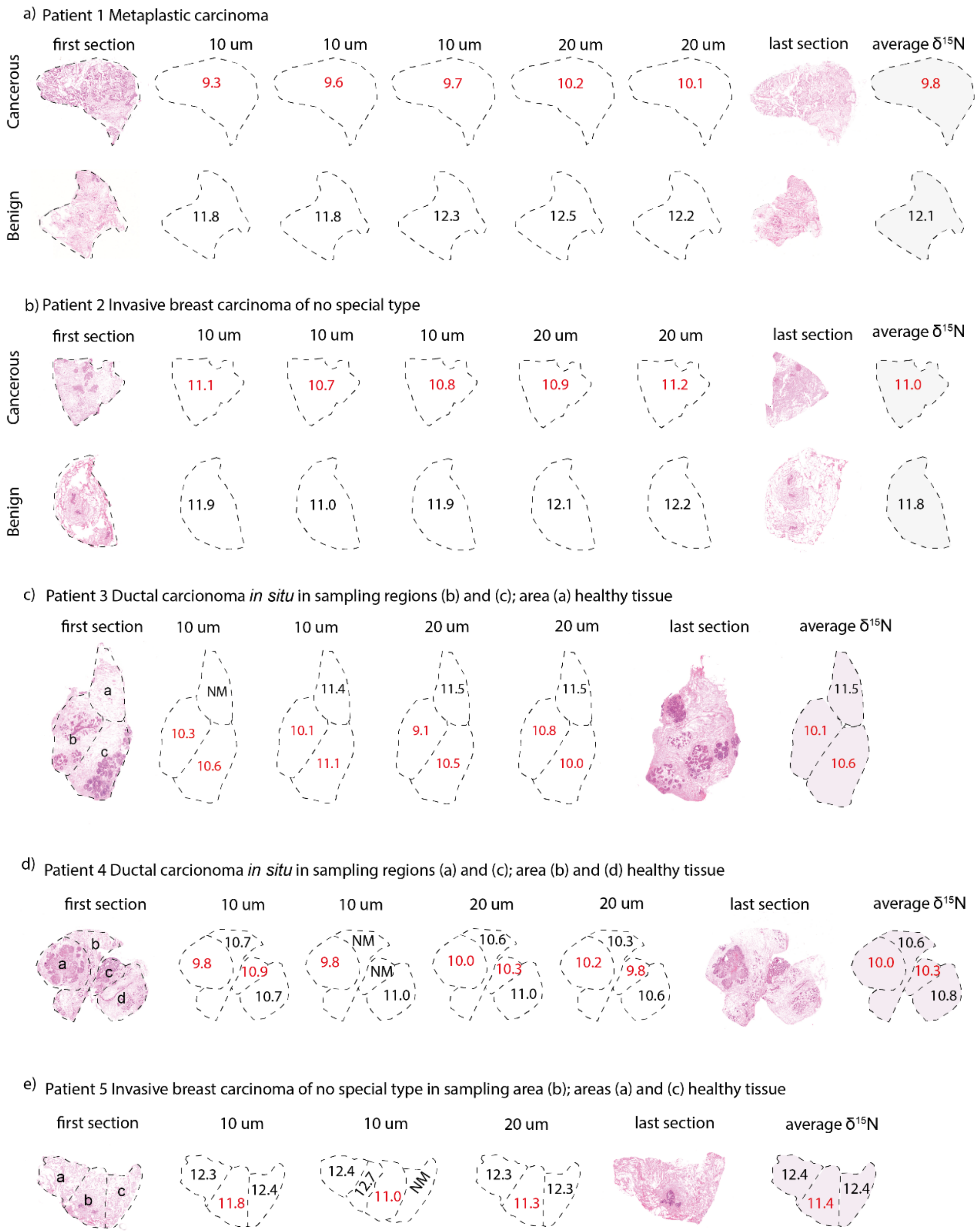


***SI Fig. S2:*** *Breast cryostat sections of patients 1 to 5. In panels a) and b) top part is cancerous tissue, bottom part is benign tissue, sampled on separate cryostat sections. In patients 3 to 4, i.e. panels c to e, one single cryostat section containing both, cancerous and benign tissue was sampled. The δ$^{15}$N (‰ vs. air) of each cryostat sections is indicated in red writing for cancerous sections/areas and in black for benign sections/areas. On the right of each panel, the average δ$^{15}$N (‰ vs. air) of all sections is indicated.*

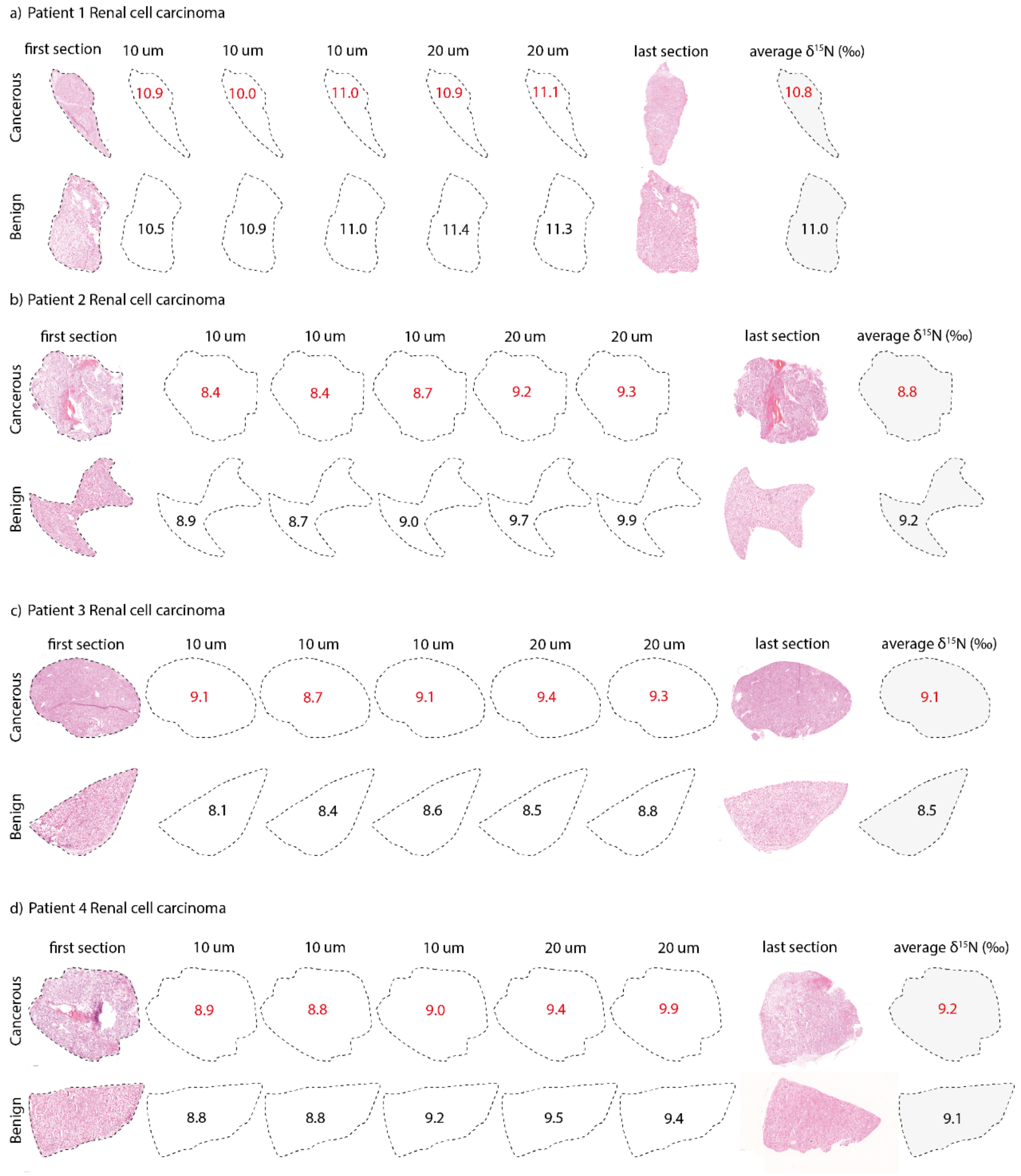


***SI Fig. S3***: *Kidney cryostat sections of patients 1 to 4, top of each panel is cancerous tissue, bottom of each panel is benign tissue. The $\delta^{15}N$ (‰ vs. air) of each cryostat sections is indicated in red writing for cancerous sections and in black for benign sections. On the right of each panel, the average $\delta^{15}N$ (‰ vs. air) of all sections is indicated.*

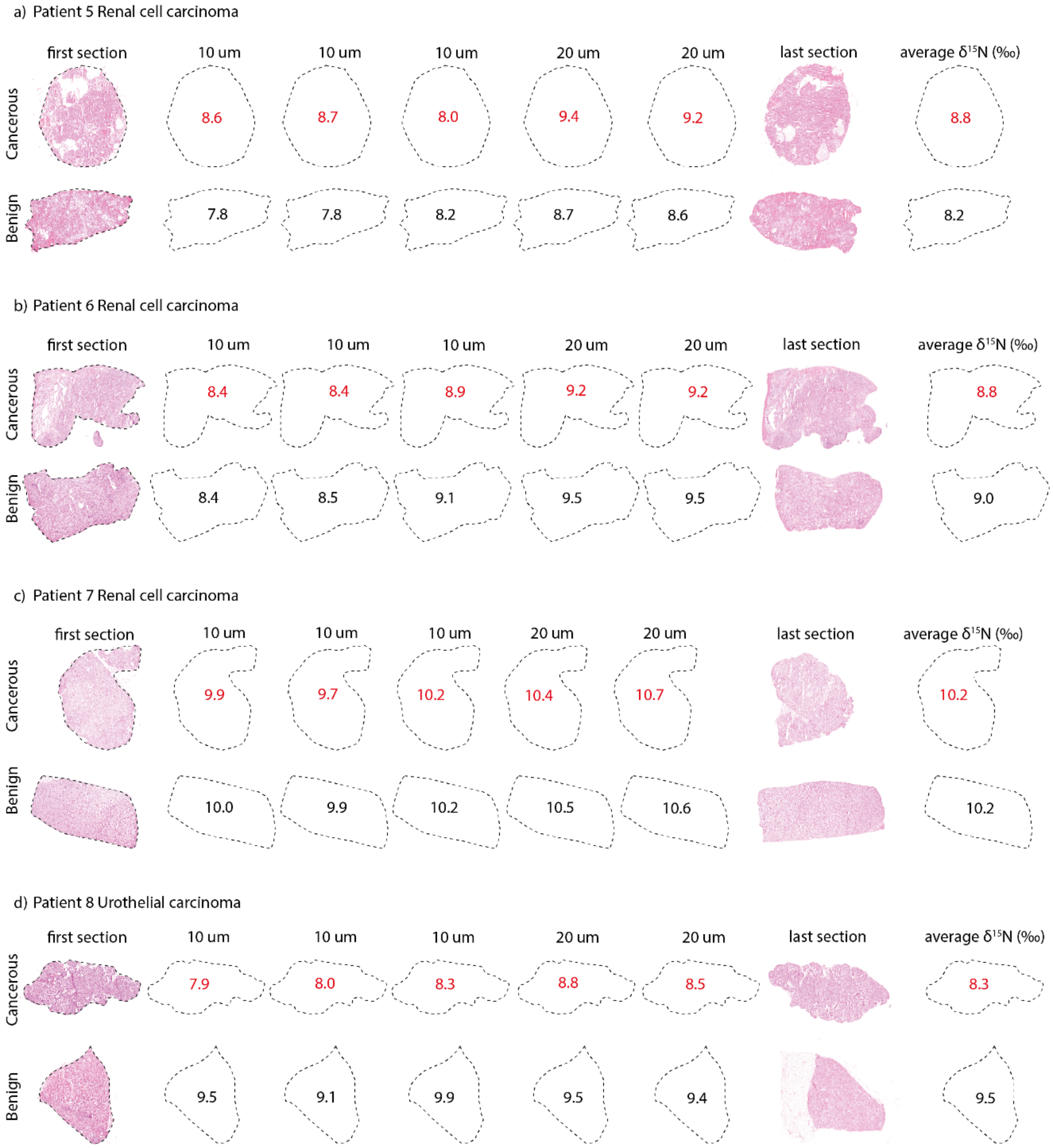


***SI Fig. S4***: *Kidney cryostat sections of patients 5 to 8, top of each panel is cancerous tissue, bottom of each panel is benign tissue. The $\delta^{15}N$ (‰ vs. air) of each cryostat sections is indicated in red writing for cancerous sections and in black for benign sections. On the right of each panel, the average $\delta^{15}N$ (‰ vs. air) of all sections is indicated.*

## 2. N isotopes analysis of bulk fine needle- and micro-biopsies

As part of this study we have also investigated the $\delta^{15}N_{B\text{-}C}$ of “bulk” surgical biopsies (SI Figs. S5 and S6). We analyzed fine needle aspiration (FNA) biopsies and scalpel-taken micro-biopsies of a few hundred micrograms taken in different parts of larger frozen surgical tissue biopsies. In this case, the histopathological characterization of the different biopsies was performed in a single cryostat section per biopsy and compared to the multiple N isotopic analyses of FNA biopsies and micro-biopsies performed in different parts of the large biopsies. The purpose of this approach was to assess the heterogeneity of the large biopsies and to evaluate if it can explain the ambiguous results obtained by previous studies that performed N isotopic measurements without histopathological control of the number of tumor cells.

Surgical biopsies were obtained from the tissue bank of the Institute of Pathology of the University Hospital of Lausanne (CHUV), Switzerland (CER-VD Project ID 2020-02064). Cryostat sections for histopathological assessment were prepared as described in the main manuscript. Fine needle aspiration (FNA) biopsies and "scalpel-based" micro-biopsies were directly taken from the freshly-frozen surgical tissue biopsies stored in Tissue-Tek® O.C.T.™ compound (OCT) at -80 °C. The tissue samples were thawed and rinsed with high-purity water before processing. For FNA biopsies, thawed tissues were sampled with a 25 G needle attached to a 10 ml syringe, the tissue biopsy was punctured, and cells and fluid were drawn up by creating a vacuum in the syringe. The sample was then transferred directly into a vial by compressing the syringe plunger and expelling the material. Scalpel-based micro-biopsies were taken by cutting off a few hundred micrograms of tissue with a scalpel. SI Tables S5 and S6 report the measurement of $\delta^{15}N$ associated with the histopathological characterization of tissues from all FNA biopsies and micro-biopsies.

Micro-biopsies were sampled in 7 lung (patients 1-7) and 12 breast cancer patients (patients 6-17). FNA biopsies were performed on 7 lung cancer patients (patients 1-7) and on 9 breast cancer patients (patients 6-13 and 15). Two to three micro-biopsies and FNA biopsies per patient were measured for their nitrogen isotopic composition. $\delta^{15}N_{B\text{-}C}$ values obtained for our multiple FNA and micro-biopsies were compared with the percentage of tumor cells obtained with

a microscopic assessment from a single cryostat section taken in a different part of the larger biopsy (i.e. the standard histopathological method in cancer diagnosis, SI Tables S4 and S5).

First, we assessed if tumor samples could be identified based on a statistically significant difference ($p \leq 0.05$) between the $\delta^{15}N$ of benign tissue and the suspected cancerous sample. Since no significant difference was observed between FNA and micro-biopsies, the measurements were combined to estimate the average and standard deviation of the suspected benign and cancerous tissue. Most of the lung cancerous tissue showed a lower mean $\delta^{15}N$ value than the corresponding benign tissue (SI Fig. S5). In 6 out of 7 lung cancer biopsies the $\delta^{15}N$ difference between the two tissues was statistically significant ($p \leq 0.05$). In patient 5, the difference was not statistically significant ($p = 0.09$), due to a higher $\delta^{15}N$ variability of the benign tissue; currently, we have no histopathological explanation for this variability. Results obtained from breast cancer biopsies were more variable, with higher standard deviations among analytical replicates, particularly in the benign tissues (SI Figure S6). Four cases of breast cancer, patients 14-17 (last column of SI Fig. S6), did not show the expected tendency of lower $\delta^{15}N$ in cancerous tissue. Histological analysis revealed only small regions of tumor in those samples (SI Table S5), which were probably missed by the subsampling for the $\delta^{15}N$ assay. Additionally, biopsies of patients 14-17 were composed of highly adipose tissue, which are not the benign tissue equivalent of the cancerous tissue and might exhibit a different $\delta^{15}N$ baseline. If breast cancer patients 14-17 are excluded for the reasons described above, from the remaining 8 breast cancer biopsies 5 showed a statistically significant ($p \leq 0.05$) lower $\delta^{15}N$ value than the corresponding benign tissue (SI Fig. S6, open dark pink and grey circles).

Second, we use the slope of the correlation between $\delta^{15}N_{\mathbf{B\text{-}C}}$ and the percentage of cancerous cells obtained in cryostat sections (Figs. 2d and e) to provide an independent estimate of tumor cell density (TCD) in our FNA and micro-biopsies and compare the results to those obtained with the standard microscopic assessment (SI Figs. S5 and S6, right part of each figure panel). Higher $\delta^{15}N$ heterogeneity in either the benign or cancerous tissue in a given sample yields higher uncertainty in the calculated TCD. Thus, the error bars in the estimated TCD provide an estimate of the heterogeneity of the tissue biopsies. The estimates of calculated TCD were

compared with those obtained with the standard microscopic assessment by the pathologist done on a single HE-stained section that had been used to select the surgical biopsies for our study (SI Figs. S5 and S6, right window, black open circles; information in SI Tables S4 and S5). In general, the two approaches showed reasonable agreement. For lung cancer, in 5 out of 7 cases, the microscopic estimate fit within the propagated uncertainty of the $\delta^{15}N$-based estimate. For breast cancer, 9 out of 12 cases fit. Nevertheless, differences between these two approaches for estimating tumor percentage are to be expected. The tumor distribution of the surgical biopsies likely varied importantly with depth, and the visual characterization only captures a tumor cell density on a single section through the biopsy. In addition, in breast tissue samples, the variable occurrence of adipose tissue likely added to the isotopic variation. Thus, we suggest that the $\delta^{15}N$-based estimate, which is based on the analysis of multiple measurements, may provide a better approximation of the average TCD in the biopsy. In any case, the large variability observed in our measurements across different patients and cancer types illustrates the importance of taking into account tissue heterogeneity when interpreting isotopic results. In this sense, our results illustrate the challenges and limitations of N isotopic measurements performed without histopathological control of the benign and cancerous tissue analyzed. This provides an explanation for the ambiguous results obtained in previous studies that measured N isotopic differences in "bulk" human biopsies without precise histopathological control of the sample analyzed.

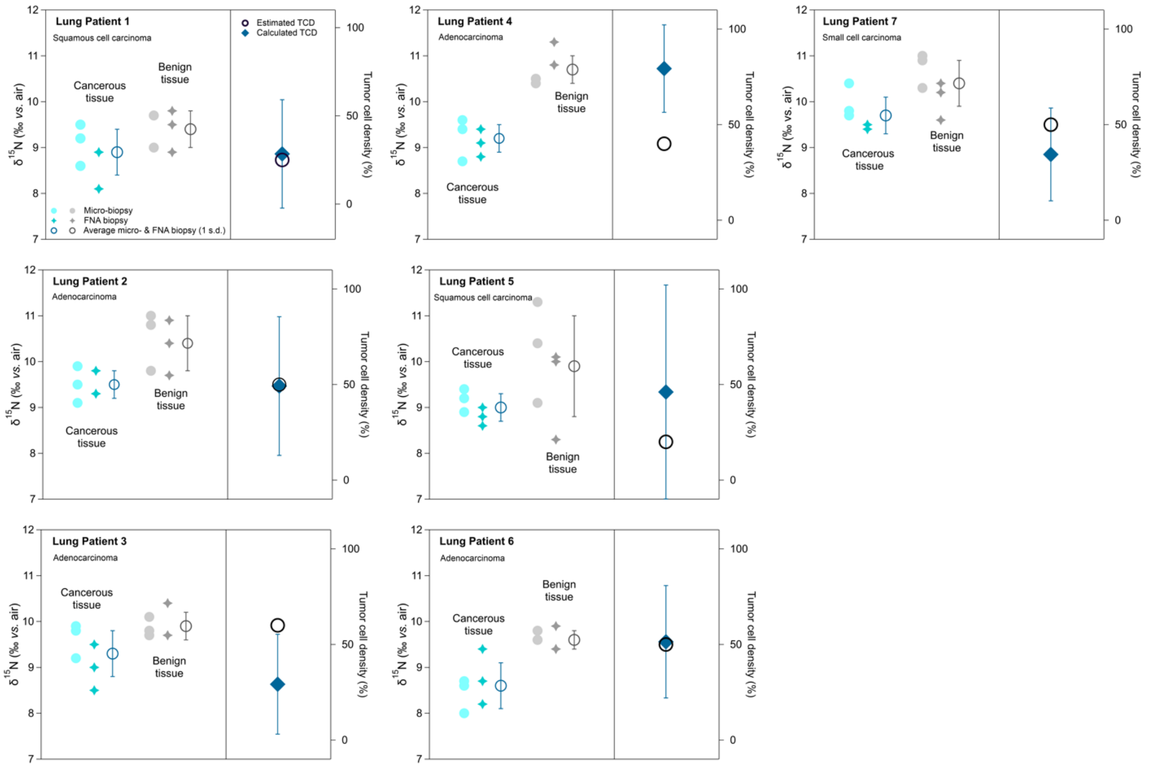


***SI Figure S5:*** *Individual $\delta^{15}N$ data from micro-biopsies (light turquoise and light grey circles) and FNA biopsy (dark turquoise and dark grey diamonds) of benign and cancerous lung tissue samples (n=3 per tissue type; except n=4 for benign tissue of patient 3). Dark turquoise and dark grey open circle: Average $\delta^{15}N$ (± 1 s.d.) from both sampling efforts (n=6 per tissue type; except n=7 for benign tissue of patient 3). For each patient, difference between benign and cancerous tissue was determined and TCD was calculated based on the coefficient values defined in Figure 1 panel d (dark turquoise diamond in the right window of each panel). The black open circle in the right window shows the tumor cell density as estimated by the pathologist on a randomly in the biopsy taken for initial biopsy selection. The legend from the first panel (patient 1) applies to all other panels (patients 2-7).*

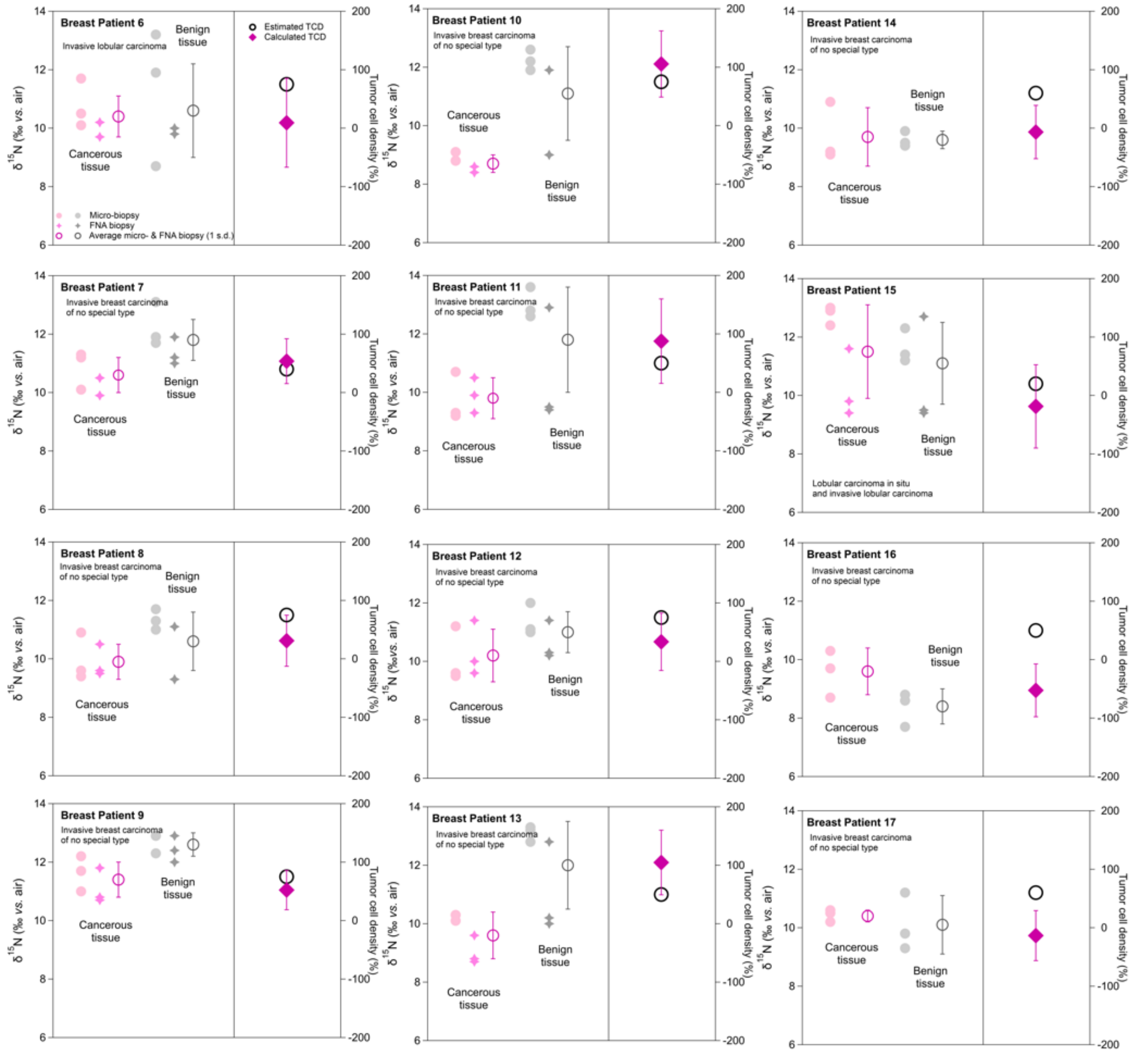


***SI Figure S6:*** *Individual δ¹⁵N data from micro-biopsies (light pink and light grey circles, n=3) and needle biopsy (pink and grey diamonds, n=3) of benign and cancerous breast tissue samples. Dark pink and dark grey open circles: Average δ¹⁵N (± 1 s.d.) from both sampling efforts (n=6 per tissue type, except n=3 for patients 14, 16 and 17). For each patient, difference between benign and cancerous tissue was determined and TCD was calculated based on the coefficient values defined in Figure 1 e (dark pink diamond in the right window of each panel). The black open circle shows the tumor cell density as estimated by the pathologist on a randomly in the biopsy taken for initial biopsy selection. The legend from the first panel (patient 6) applies to all other panels (patients 6-17).*

## 3. Description SI Tables

SI Tables S1-S5: All tables include patient number, tissue type, gender and age, $\delta^{15}N$ (1.s.d.), tumor cell percentage as quantified by pathologist, pathological description of cryostat section, microscopic images of cryostat sections.

**SI Table S1 Lung cancer patients**

| Patient Nr. | Tissue type | Gender | Age | $\delta^{15}N$ | 1.s.d. | Tumor cell density (%) | Description | Cancerous tissue (first section) | Cancerous tissue (last section) | Benign tissue (first section) | Benign tissue (last section) |
|---|---|---|---|---|---|---|---|---|---|---|---|
| **1** | Cancerous | F | 80 | 8.6 | 0.3 | 70 % tumor cells, homogeneously distributed | INVASIVE SQUAMOUS CELL CARCINOMA | 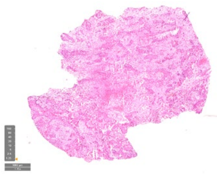 | 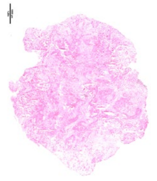 | 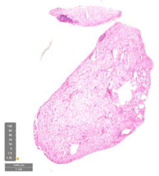 | 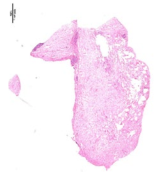 |
| | Benign | | | 10.1 | 0.2 | NA | Normal lung tissue | | | | |
| **2** | Cancerous | M | 72 | 9.2 | 0.4 | 50 % tumor cells, homogeneously distributed | INVASIVE ADENOCARCINOMA | 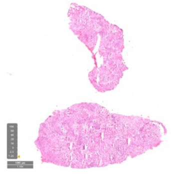 | 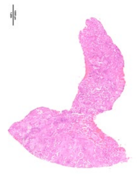 | 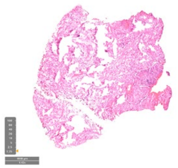 | 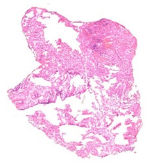 |
| | Benign | | | 10.2 | 0.4 | NA | Normal lung tissue | | | | |
| **3** | Cancerous | M | 66 | 9.4 | 0.4 | 50 % tumor cells, homogeneously distributed | INVASIVE ADENOCARCINOMA | 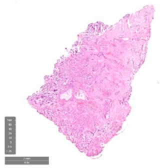 | 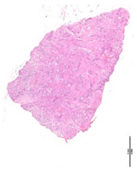 | 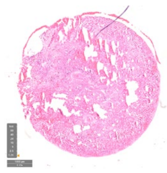 | 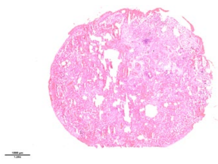 |
| | Benign | | | 10.3 | 0.2 | NA | Normal lung tissue | | | | |
| **4** | Cancerous | F | 55 | 9.0 | 0.3 | 35 % tumor cells, homogeneously distributed | INVASIVE ADENOCARCINOMA | 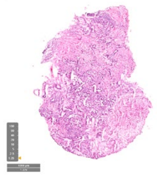 | 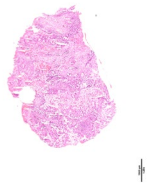 | 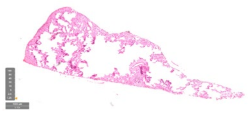 | 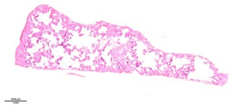 |
| | Benign | | | 9.6 | 0.3 | NA | Normal lung tissue | | | | |

**SI Table S2 Breast cancer patients**

| Patient Nr. | Cancer/tissue type | Gender | Age | $\delta^{15}N$ | 1.s.d. | Tumor cell density (%) | Description | Cancerous tissue (first section) | Cancerous tissue (last section) | Benign tissue (first section) | Benign tissue (last section) |
|---|---|---|---|---|---|---|---|---|---|---|---|
| 1 | Cancerous | F | 51 | 9.8 | 0.4 | 70 % tumor cells, homogeneously distributed | METAPLASTIC CARCINOMA | 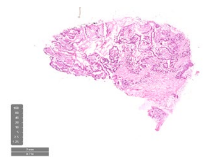 | 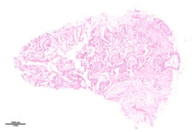 | 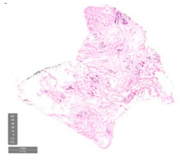 | 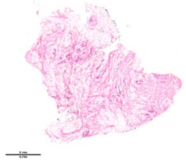 |
| | Benign | | | 12.1 | 0.3 | NA | Normal breast tissue | | | | |
| 2 | Cancerous | F | 55 | 11.0 | 0.2 | 65 % tumor cells, homogeneously distributed | INVASIVE BREAST CARCINOMA OF NO SPECIAL TYPE | 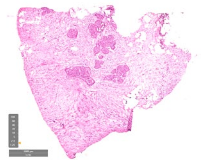 | 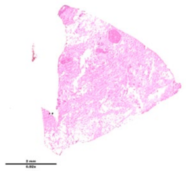 | 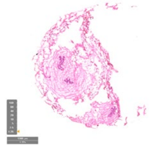 | 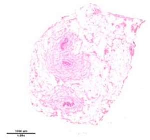 |
| | Benign | | | 11.8 | 0.5 | NA | Normal breast tissue | | | | |
| 3 | Mix cancerous/benign | F | 46 | See SI Figure 2 | | | DUCTAL CARCINOMA IN SITU | 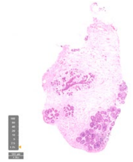 | 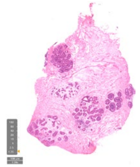 | | |
| 4 | Mix cancerous/benign | F | 40 | See SI Figure 2 | | | DUCTAL CARCINOMA IN SITU | 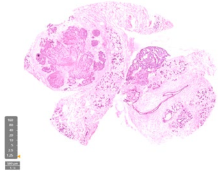 | 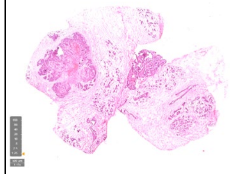 | | |
| 5 | Mix cancerous/benign | F | 37 | See SI Figure 2 | | | INVASIVE BREAST CARCINOMA OF NO SPECIAL TYPE | 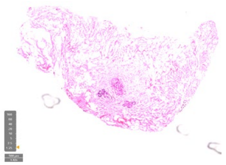 | 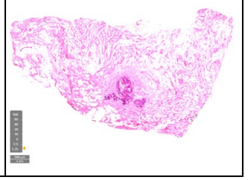 | | |

**SI Table S3 Kidney cancer patients**

| Patient Nr. | Tissue type | Gender | Age | $\delta^{15}N$ | 1.s.d. | Tumor cell density (%) | Description | Cancerous tissue (first section) | Cancerous tissue (last section) | Benign tissue (first section) | Benign tissue (last section) |
|---|---|---|---|---|---|---|---|---|---|---|---|
| 1 | Cancerous | M | 68 | 10.8 | 0.4 | 90 % tumor cells, homogeneously distributed | CLEAR CELL RENAL CELL CARCINOMA | 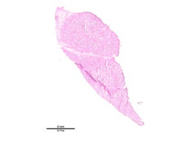 | 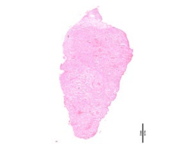 | 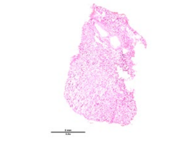 | 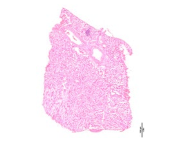 |
| | Benign | | | 11.0 | 0.4 | NA | Normal kidney tissue | | | | |
| 2 | Cancerous | M | 63 | 8.8 | 0.4 | 90 % tumor cells, homogeneously distributed | CLEAR CELL RENAL CELL CARCINOMA | 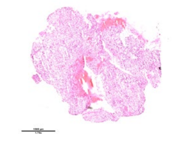 | 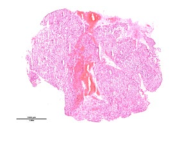 | 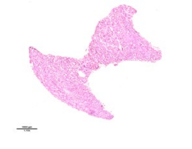 | 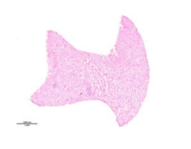 |
| | Benign | | | 9.2 | 0.5 | NA | Normal kidney tissue | | | | |
| 3 | Cancerous | F | 54 | 9.1 | 0.3 | 90 % tumor cells, homogeneously distributed | CLEAR CELL RENAL CELL CARCINOMA | 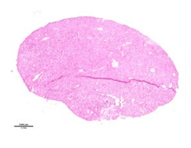 | 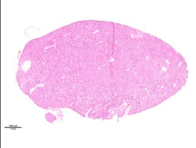 | 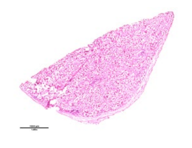 | 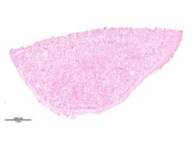 |
| | Benign | | | 8.5 | 0.2 | NA | Normal kidney tissue | | | | |
| 4 | Cancerous | F | 43 | 9.2 | 0.5 | 80 % tumor cells, homogeneously distributed | CLEAR CELL RENAL CELL CARCINOMA | 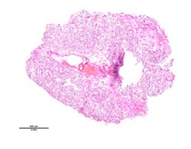 | 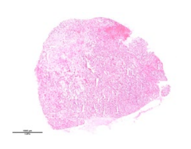 | 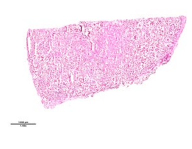 | 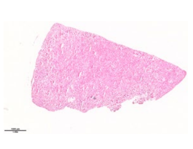 |
| | Benign | | | 9.1 | 0.3 | NA | Normal kidney tissue | | | | |
| 5 | Cancerous | M | 61 | 8.8 | 0.6 | 90 % tumor cells, homogeneously distributed | PAPILLARY RENAL CELL CARCINOMA | 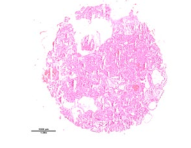 | 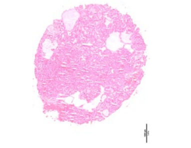 | 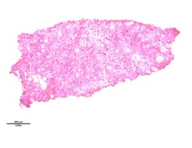 | 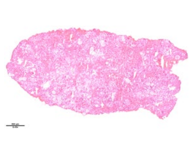 |
| | Benign | | | 8.2 | 0.4 | NA | Normal kidney tissue | | | | |
| 6 | Cancerous | M | 59 | 8.8 | 0.4 | 85 % tumor cells, homogeneously distributed | CLEAR CELL RENAL CELL CARCINOMA | 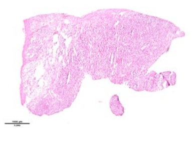 | 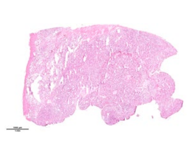 | 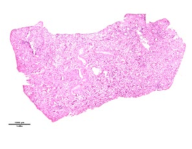 | 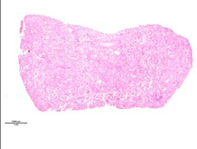 |
| | Benign | | | 9.0 | 0.5 | NA | Normal kidney tissue | | | | |
| 7 | Cancerous | M | 78 | 10.2 | 0.4 | 90 % tumor cells, homogeneously distributed | CLEAR CELL RENAL CELL CARCINOMA | 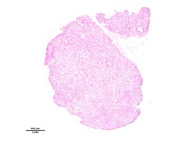 | 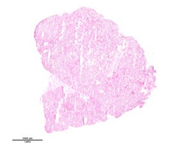 | 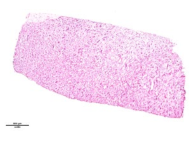 | 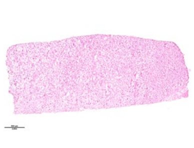 |
| | Benign | | | 10.2 | 0.3 | NA | Normal kidney tissue | | | | |
| 8 | Cancerous | M | 73 | 8.3 | 0.4 | 90 % tumor cells, homogeneously distributed | UROTHELIAL CARCINOMA | 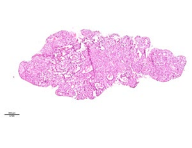 | 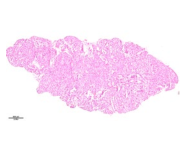 | 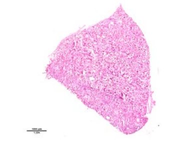 | 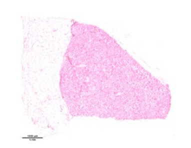 |
| | Benign | | | 9.5 | 0.3 | NA | Normal kidney tissue | | | | |

SI Table S4 Bulk biopsies lung cancer patients

| Patient Nr. | Tissue type | Gender | Age | $\delta^{15}N$ | 1.s.d. | Tumor cell density (%) | Description | Cancerous tissue | Benign tissue |
|---|---|---|---|---|---|---|---|---|---|
| **1** | Cancerous | F | 80 | 8.9 | 0.5 | 25 % tumor cells | INVASIVE SQUAMOUS CELL CARCINOMA | 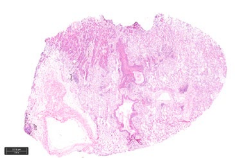 | 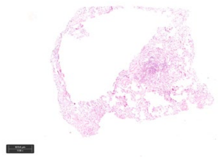 |
| | Benign | | | 9.4 | 0.4 | NA | Normal lung tissue, focal inflammation | | |
| **2** | Cancerous | M | 72 | 9.5 | 0.3 | 50% tumor cells | ADENOCARCINOMA | 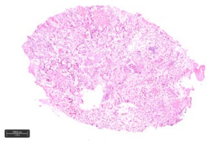 | 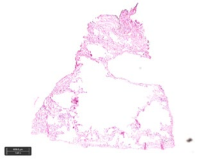 |
| | Benign | | | 10.4 | 0.6 | NA | Normal lung tissue | | |
| **3** | Cancerous | M | 66 | 9.3 | 0.5 | 60% tumor cells | ADENOCARCINOMA | 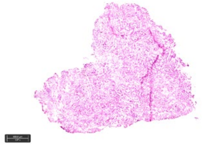 | 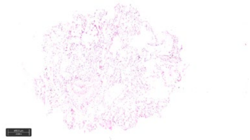 |
| | Benign | | | 9.9 | 0.3 | NA | Normal lung tissue | | |
| **4** | Cancerous | F | 55 | 9.2 | 0.3 | 40% tumor cells | ADENOCARCINOMA | 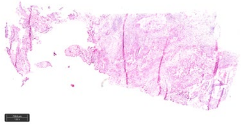 | 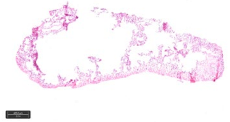 |
| | Benign | | | 10.7 | 0.3 | NA | Normal lung tissue | | |
| **5** | Cancerous | M | 71 | 9.0 | 0.3 | 20% tumor cells | SQUAMOUS CELL CARCINOMA | 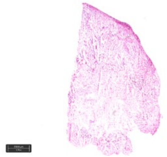 | 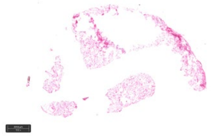 |
| | Benign | | | 9.9 | 1.1 | NA | Normal lung tissue | | |
| **6** | Cancerous | M | 82 | 8.6 | 0.5 | 50% tumor cells | ADENOCARCINOMA | 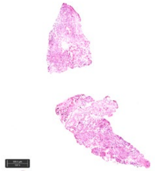 | 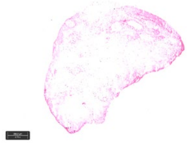 |
| | Benign | | | 9.6 | 0.2 | NA | Normal lung tissue | | |
| **7** | Cancerous | F | 65 | 9.7 | 0.4 | 50 % tumor cells | SMALL CELL CARCINOMA | 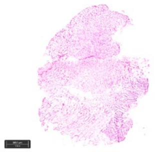 | 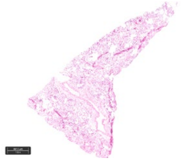 |
| | Benign | | | 10.4 | 0.5 | NA | Normal lung tissue | | |

SI Table S5 Bulk biopsies breast cancer patients

| Patient Nr. | Tissue type | Gender | Age | $\delta^{15}N$ | 1.s.d. | Tumor cell density (%) | Description | Cancerous tissue | Benign tissue |
|---|---|---|---|---|---|---|---|---|---|
| **6** | Cancerous | F | 48 | 10.4 | 0.7 | 75% tumor cells | INVASIVE LOBULAR CARCINOMA | 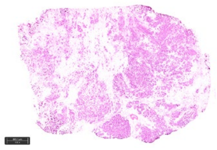 | 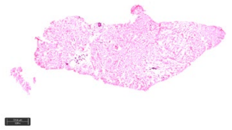 |
| | Benign | | | 10.6 | 1.6 | NA | Normal breast tissue | | |
| **7** | Cancerous | F | 49 | 10.6 | 0.6 | 40% tumor cells | INVASIVE BREAST CARCINOMA OF NO SPECIAL TYPE | 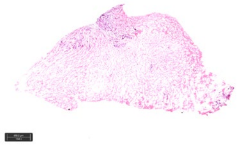 | 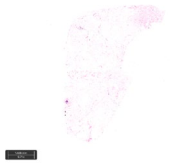 |
| | Benign | | | 11.8 | 0.7 | NA | Normal breast tissue | | |
| **8** | Cancerous | F | 35 | 9.9 | 0.6 | 75% tumor cells | INVASIVE BREAST CARCINOMA OF NO SPECIAL TYPE | 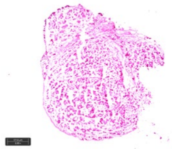 | 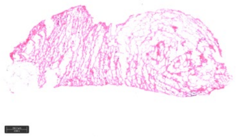 |
| | Benign | | | 10.6 | 1.0 | NA | Normal breast tissue | | |
| **9** | Cancerous | F | 76 | 11.4 | 0.6 | 75% tumor cells | INVASIVE BREAST CARCINOMA OF NO SPECIAL TYPE | 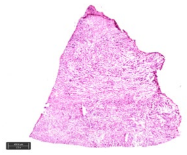 | 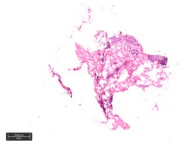 |
| | Benign | | | 12.6 | 0.4 | NA | Normal breast tissue | | |
| **10** | Cancerous | F | 73 | 8.7 | 0.3 | 75% tumor cells | INVASIVE BREAST CARCINOMA OF NO SPECIAL TYPE | 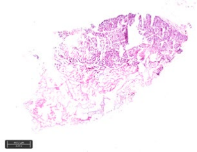 | 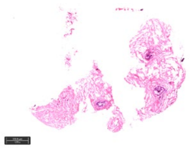 |
| | Benign | | | 11.1 | 1.6 | NA | Normal breast tissue | | |
| **11** | Cancerous | F | 68 | 9.8 | 0.7 | 50% tumor cells | INVASIVE BREAST CARCINOMA OF NO SPECIAL TYPE | 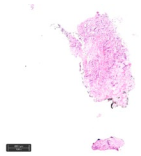 | 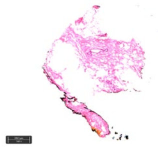 |
| | Benign | | | 11.8 | 1.8 | NA | Normal breast tissue | | |

| | | | | | | | | | |
|---|---|---|---|---|---|---|---|---|---|
| **12** | Cancerous | F | 28 | 10.2 | 0.9 | 75% tumor cells | Invasive breast carcinoma of no special type | 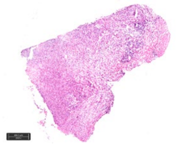 | 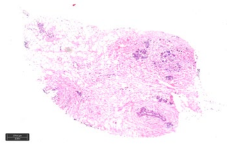 |
| | Benign | | | 11.0 | 0.7 | NA | Normal breast tissue | | |
| **13** | Cancerous | F | 38 | 9.6 | 0.8 | 50% tumor cells | INVASIVE BREAST CARCINOMA OF NO SPECIAL TYPE | 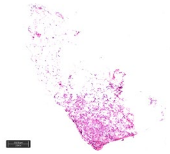 | 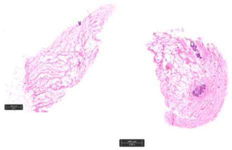 |
| | Benign | | | 12.0 | 1.5 | NA | Normal breast tissue | | |
| **14** | Cancerous | F | 93 | 9.7 | 1.0 | 60% tumor cells | INVASIVE BREAST CARCINOMA OF NO SPECIAL TYPE | 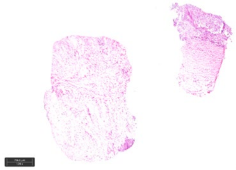 | 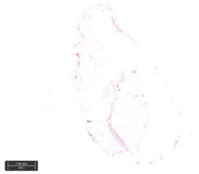 |
| | Benign | | | 9.6 | 0.3 | NA | Normal breast tissue (adipose) | | |
| **15** | Cancerous | F | 57 | 11.5 | 1.6 | 20% tumor cells | LOBULAR CARCINOMA IN SITUT AND INVASIVE LOBULAR CARCINOMA | 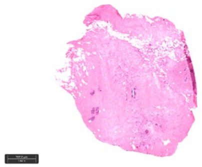 | 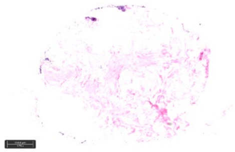 |
| | Benign | | | 11.1 | 1.4 | NA | Normal breast tissue (adipose) | | |
| **16** | Cancerous | F | 36 | 9.6 | 0.8 | 50% tumor cells | INVASIVE BREAST CARCINOMA OF NO SPECIAL TYPE | 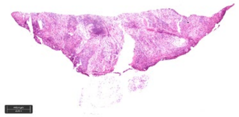 | 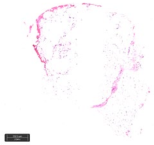 |
| | Benign | | | 8.4 | 0.6 | NA | Normal breast tissue (adipose) | | |
| **17** | Cancerous | F | 77 | 10.4 | 0.2 | 60% tumor cells | INVASIVE BREAST CARCINOMA OF NO SPECIAL TYPE | 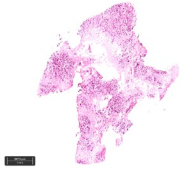 | 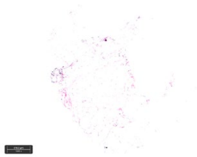 |
| | Benign | | | 10.1 | 1.0 | NA | Normal breast tissue (adipose) | | |
